# Rational Design of Live-Attenuated Vaccines against Genome-Reduced Pathogens

**DOI:** 10.1128/spectrum.03776-22

**Published:** 2022-12-01

**Authors:** Sayaka Nishikawa, Yohsuke Ogawa, Kazumasa Shiraiwa, Rieko Nozawa, Momoko Nakayama, Masahiro Eguchi, Yoshihiro Shimoji

**Affiliations:** a National Institute of Animal Health, National Agriculture and Food Research Organization (NARO), Tsukuba, Japan; b Research Institute for Biomedical Sciences, Tokyo University of Science, Noda, Chiba, Japan; Texas A&M University

**Keywords:** *Erysipelothrix rhusiopathiae*, genome-reduced bacteria, metabolic adaptation, vaccine design

## Abstract

To develop safe and highly effective live vaccines, rational vaccine design is necessary. Here, we sought a simple approach to rationally develop a safe attenuated vaccine against the genome-reduced pathogen Erysipelothrix rhusiopathiae. We examined the mRNA expression of all conserved amino acid biosynthetic genes remaining in the genome after the reductive evolution of *E. rhusiopathiae*. Reverse transcription-quantitative PCR (qRT-PCR) analysis revealed that half of the 14 genes examined were upregulated during the infection of murine J774A.1 macrophages. Gene deletion was possible only for three proline biosynthesis genes, *proB*, *proA*, and *proC*, the last of which was upregulated 29-fold during infection. Five mutants bearing an in-frame deletion of one (Δ*proB*, Δ*proA*, or Δ*proC* mutant), two (Δ*proBA* mutant), or three (Δ*proBAC* mutant) genes exhibited attenuated growth during J774A.1 infection, and the attenuation and vaccine efficacy of these mutants were confirmed in mice and pigs. Thus, for the rational design of live vaccines against genome-reduced bacteria, the selective targeting of genes that escaped chromosomal deletions during evolution may be a simple approach for identifying genes which are specifically upregulated during infection.

**IMPORTANCE** Identification of bacterial genes that are specifically upregulated during infection can lead to the rational construction of live vaccines. For this purpose, genome-based approaches, including DNA microarray analysis and IVET (*in vivo* expression technology), have been used so far; however, these methods can become laborious and time-consuming. In this study, we used a simple *in silico* approach and showed that in genome-reduced bacteria, the genes which evolutionarily remained conserved for metabolic adaptations during infection may be the best targets for the deletion and construction of live vaccines.

## INTRODUCTION

Vaccination is the most effective strategy for disease control and prevention. Despite the great success of vaccination in human and animal health, several challenges still remain in vaccine development. Novel vaccine technologies, including DNA, RNA, and live-vector vaccines, have already been applied in veterinary medicine (1). Nevertheless, for the livestock industry, attenuated live vaccines are preferable because they are produced at a low cost and usually sufficient to induce long-term, sometimes lifelong protection with a single dose (2). Thus far, live vaccines have traditionally been prepared via empirical attenuation approaches such as serial passaging and chemical mutagenesis (3); however, achieving an optimal balance of attenuation and immunogenicity is very difficult, and there is a risk of reversion to pathogenicity and residual virulence (2–4). Hence, to develop safe and highly protective live vaccines, rational vaccine design through modern biological approaches is mandatory.

Bacterial pathogens tightly regulate gene expression to survive in various harsh environments and successfully establish infection in hosts. The identification of genes that are specifically upregulated during infection can aid in the construction of safe and effective live-attenuated vaccines. For this purpose, genome-based approaches, including DNA microarray analysis and *in vivo* expression technology (IVET), have been used to successfully identify *in vivo*-induced genes in many different bacteria (5–10). Thus, the utilization of genome-mining approaches for identifying genes that are upregulated during infection has been extensively reviewed; however, it has not been reported or widely recognized that in genome-reduced pathogens, the “reverse vaccinology” approach facilitates the rapid and easy identification of genes that are specifically upregulated *in vivo*.

The Gram-positive bacterium Erysipelothrix rhusiopathiae is an intracellular pathogen that causes erysipelas in many species of animals and erysipeloid in humans. It is best recognized as the causative agent of swine erysipelas, a severe disease characterized by acute septicemia or chronic arthritis and endocarditis (11). Vaccines conferring improved protection are urgently needed for the control of swine erysipelas (12), and oral vaccines that can reduce vaccination costs and stress to animals are particularly desired (13).

In this study, to develop a safe and effective oral vaccine against swine erysipelas, we used a genomic-based *in silico* approach. Whole-genome sequence analysis of the highly virulent *E. rhusiopathiae* Fujisawa strain revealed that the *E. rhusiopathiae* genome has entirely lost fatty acid biosynthetic pathways and lacks genes for the biosynthesis of many amino acids, cofactors, and vitamins, indicating reductive genome evolution (14). Despite this large-scale genome reduction, the small *E. rhusiopathiae* genome encodes nine antioxidant factors and nine phospholipases (14), showing that intracellular survival and replication are the most important properties in the pathogenicity of *E. rhusiopathiae* (15). The intracellular survival mechanisms used by *E. rhusiopathiae* are still not fully understood; however, for intracellular pathogens, the acquisition of host nutrients and metabolic adaptations to the nutritional environments within the host are fundamental virulence strategies for surviving in host animals (16–19). Based on these findings, we hypothesized that in genome-reduced pathogens evolutionarily adapted to intracellular environments, the selective targeting of genes encoding certain nutrient biosynthesis pathways may be a simple theoretical approach for the identification of *in vivo*-upregulated genes during infection.

To test our hypothesis, we focused on the genes responsible for amino acid biosynthesis. *E. rhusiopathiae* possesses complete biosynthetic pathways for seven amino acids (alanine, asparagine, glutamine, serine, cysteine, glycine, and proline) and an incomplete pathway for arginine (14). We examined the mRNA expression of all 14 genes involved in these biosynthetic pathways during the infection of murine macrophages. We further constructed gene deletion mutants for three genes involved in the proline biosynthesis pathway and investigated their attenuation and vaccine efficacy in mice and pigs.

## RESULTS

### Expression of amino acid biosynthetic genes of *E. rhusiopathiae* during macrophage infection.

**FIG 1:**
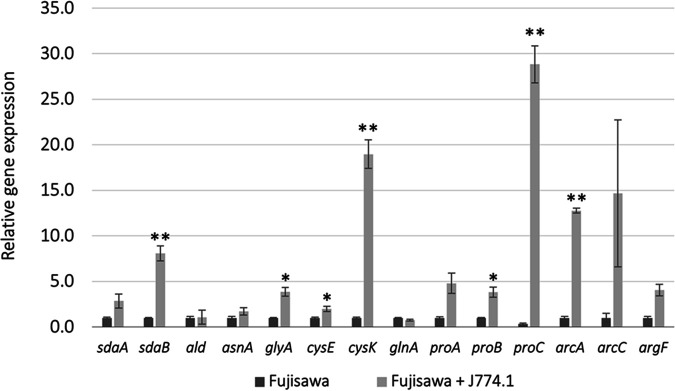
mRNA expression of amino acid biosynthetic genes during infection of murine J774.1 macrophages. Macrophages and the wild-type Erysipelothrix rhusiopathiae Fujisawa strain were incubated for 6 h. Relative expression levels of the indicated genes were determined by reverse transcription-quantitative PCR (qRT-PCR) assays and normalized to *23S rRNA* expression levels. Data are presented as the mean ± standard error of the mean (SEM) from three independent experiments. **, *P < *0.01; *, *P < *0.05 by paired *t* test, compared to data obtained from samples without macrophages. *sdaA*, l-serine dehydratase, iron-sulfur-dependent, alpha subunit (ERH_1322); *sdaB*, l-serine dehydratase, iron-sulfur-dependent, beta subunit (ERH_1323); *ald*, alanine dehydrogenase (ERH_1073); *asnA*, aspartate-ammonia ligase (ERH_1353); *glyA*, glycine hydroxymethyltransferase (ERH_1608); *cysE*, serine *O*-acetyltransferase (ERH_0421); *cysK*, cysteine synthase A (ERH_0469); *glyA*, glycine hydroxymethyltransferase (ERH_1608); *proA*, glutamate-5-semialdehyde dehydrogenase (ERH_0055); *proB*, glutamate 5-kinase (ERH_0054); *proC*, pyrroline-5-carboxylate reductase (ERH_0057); *arcA*, arginine deiminase (ERH_0795); *arcC*, carbamate kinase (ERH_0797); *argF*, ornithine carbamoyltransferase (ERH_0796).

*E. rhusiopathiae* possesses a total of 14 genes for complete and incomplete biosynthetic pathways of amino acids. Reverse transcription-quantitative PCR (qRT-PCR)-based expression analysis of all amino acid biosynthetic genes of the *E. rhusiopathiae* Fujisawa strain revealed that during 6 h of interaction with murine macrophage J774A.1 cells, seven (*sdaB*, *glyA*, *cysE*, *cysK*, *proB*, *proC*, and *arcA*) out of the 14 genes tested were significantly upregulated compared to their expression levels during growth in medium without macrophages (Fig. 1). Among the upregulated genes, very high expression levels were observed for *cysK*, *proC*, and *arcA*, whose expression levels were upregulated 19.0-, 28.8-, and 12.8-fold, respectively.

### Proliferation of proline biosynthetic gene deletion mutants during macrophage infection.

The construction of gene deletion mutants was unsuccessful for 11 genes involved in the biosynthetic pathways of seven amino acids, including alanine, asparagine, glutamine, serine, cysteine, glycine, and arginine. However, we were able to delete the genes *proB*, *proA*, and *proC*, all of which are involved in proline biosynthesis, and we constructed five mutants bearing an in-frame gene deletion of one (Δ*proB*, Δ*proA*, or Δ*proC* mutant), two (Δ*proBA* mutant), or three (Δ*proBAC* mutant) of these genes.

**FIG 2:**
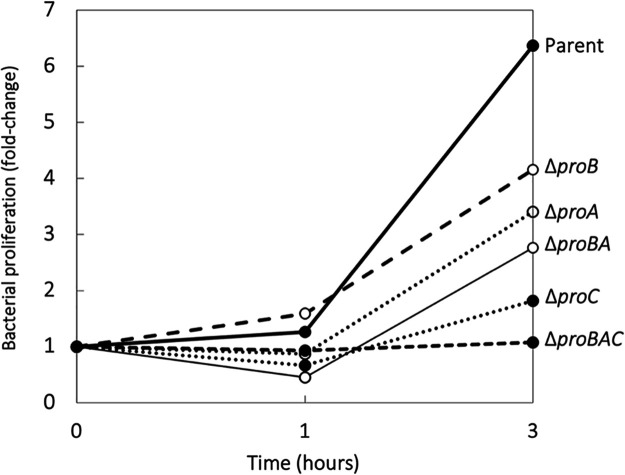
Proliferation of *E. rhusiopathiae* strains during the infection of murine J774.1 macrophages. Bacterial proliferation was expressed as a fold change normalized to the initial number of viable intracellular bacteria. The results are representative of three separate experiments.

**FIG 3:**
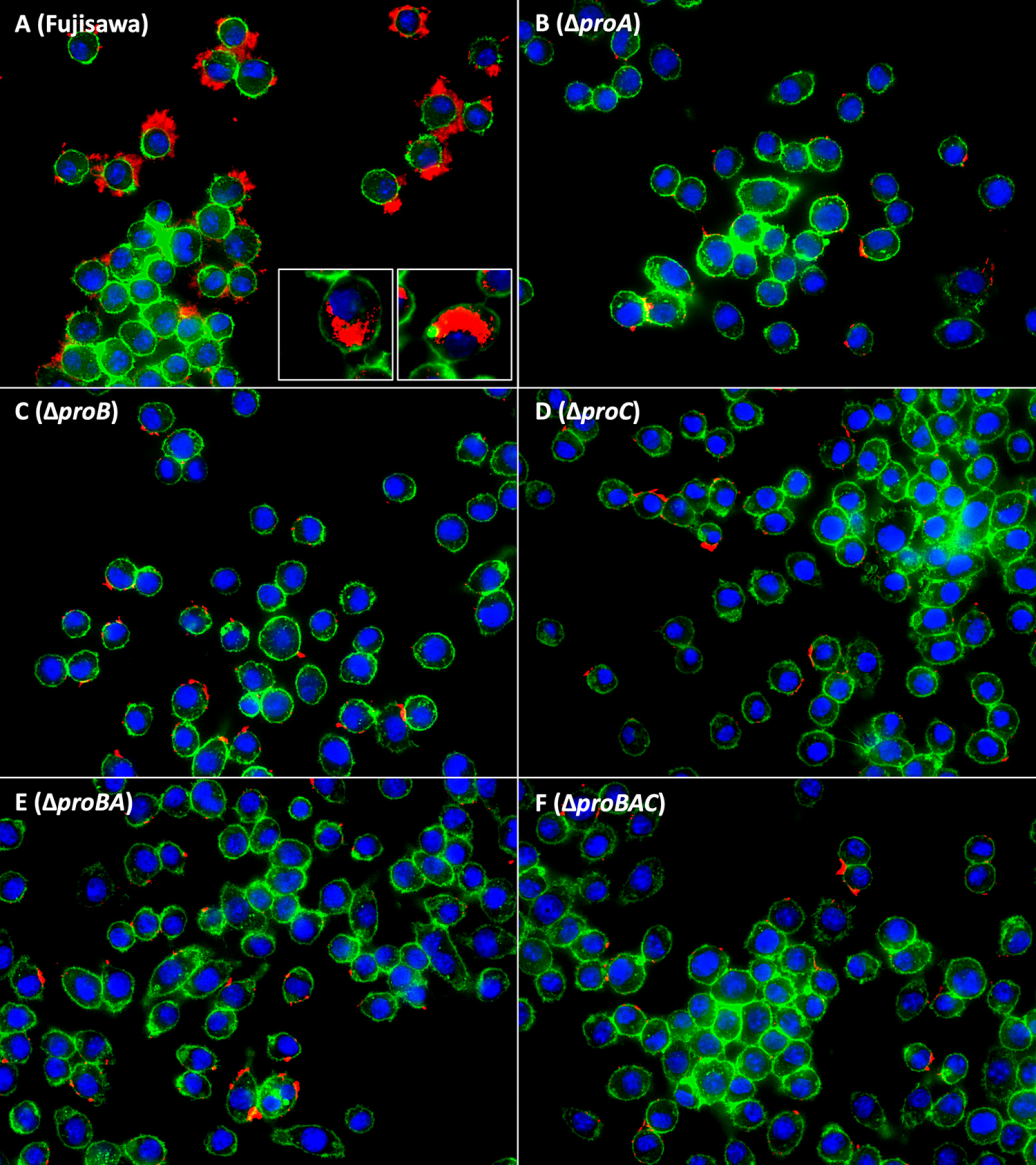
Microscopy observations of the growth of *E. rhusiopathiae* strains during infection with murine macrophages. Macrophage J774.1 cells were infected with *E. rhusiopathiae* strains at a multiplicity of infection (MOI) of 1:1 for 1 h, washed, and then incubated for 16 h. Images were taken at a magnification of 600- (A to F) or 1,000-fold (panel A, insets).

Murine macrophage J774A.1 cells were infected with either the parental wild-type strain or the gene deletion mutants, and the effect of the deletion of the proline biosynthetic genes on the proliferation ability of *E. rhusiopathiae* was examined. We observed that the proliferation rates of the gene deletion mutants varied, but those of the Δ*proC* and Δ*proBAC* strains were consistently low. The typical proliferation patterns of the mutants are shown in Fig. 2. In this experiment, after a 3-h incubation period, the parental Fujisawa strain proliferated 6.37-fold, whereas the proliferation rates of the Δ*proC* and Δ*proBAC* strains were 1.82- and 1.08-fold, respectively. In RPMI 1640 medium containing 10% fetal bovine serum (RPMI-FBS) alone, there were no differences in the numbers of live bacteria of the Fujisawa and mutant strains (Fig. S1). Microscopy observations showed that the parent Fujisawa strain proliferated extracellularly, but the proliferation of the other proline mutants was limited (Fig. 3). Importantly, intracellular proliferation within macrophages was observed only in the parental Fujisawa strain (Fig. 3A; insets).

### Growth of proline biosynthetic mutants in mice.

**FIG 4:**
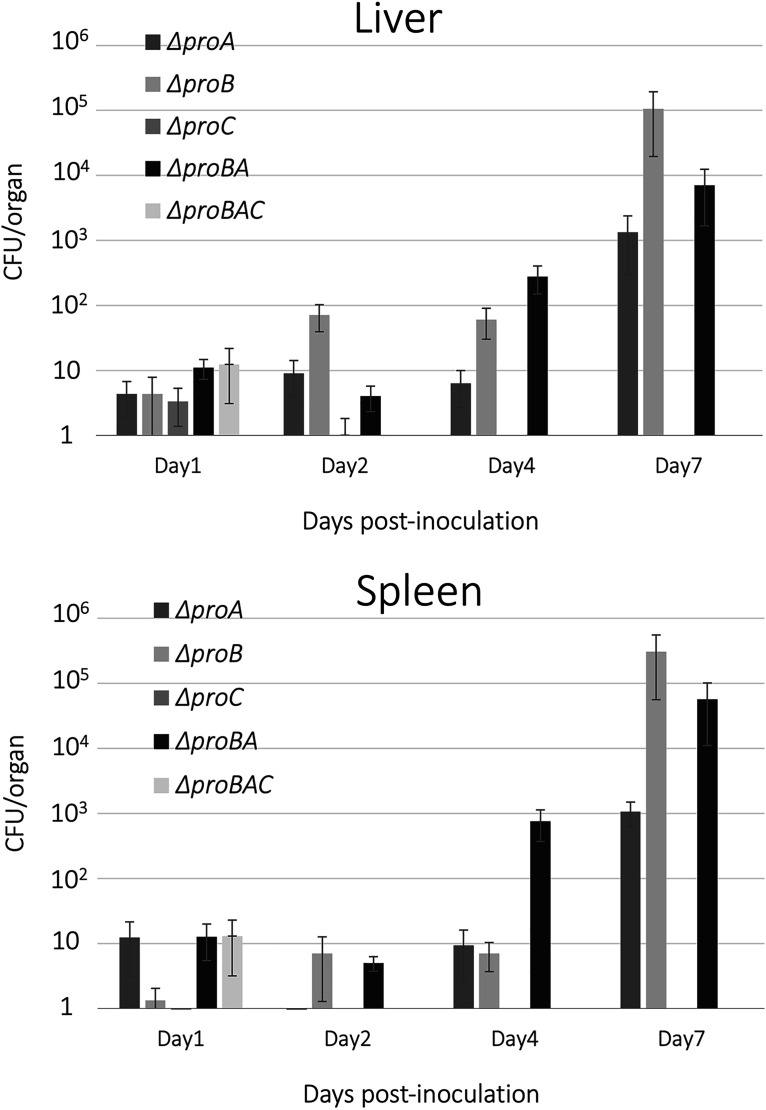
Growth of *E. rhusiopathiae* strains in mice. Data are presented as the mean ± SEM from three independent experiments. No bacteria of the Δ*proC* or Δ*proBAC* strains were recovered from livers and spleens collected on day 2 or thereafter.

In mice, virulent *E. rhusiopathiae* strains invariably cause death on the third day after infection; hence, the effect of the deletion of the proline biosynthetic genes on the virulence of the *E. rhusiopathiae* mutants was examined by estimating the numbers of bacteria grown in the livers and spleens of mice up to 7 days after infection. As shown in Fig. 4, the Δ*proC* and Δ*proBAC* strains were cleared from livers and spleens by day 2 and could not be recovered thereafter. In the Δ*proA*, Δ*proB*, and Δ *proBA* strains, the number of isolated bacteria increased with time during the observation period.

### Virulence and vaccine efficacy of gene deletion mutants in mice.

The virulence and vaccine efficacy of the proline biosynthesis mutants were analyzed in mice (Table 1). The Δ*proC* and Δ*proBAC* strains did not cause any clinical symptoms in mice, whereas Δ*proA*, Δ*proB*, and Δ*proBA* caused anorexia, depression, ruffled fur, hunched posture, arthritis, and/or conjunctivitis in 1 to 6 mice in each group at 8 to 11 days after inoculation, but all mice survived. The mice were challenged with 100 LD_50_ (50% lethal dose) of the parent Fujisawa strain, and the protective capability of the mutant strains was examined. The results showed that all mice vaccinated with the mutants were protected against lethal infection challenge.

**TABLE 1:** Virulence and vaccine efficacy of Erysipelothrix rhusiopathiae proline biosynthesis mutants in mice^*a*^

Mouse inoculation group	No. of survivors/total on day 14 after inoculation	No. of survivors/total on day 14 after challenge infection
Non-inoculated	NA	0/10
Fujisawa	0/10	NA
Δ*proA*	10/10^*b*^	10/10^*b*^
Δ*proB*	10/10^*b*^	10/10^*b*^
Δ*proC*	10/10^*b*^	10/10^*b*^
Δ*proBA*	10/10^*b*^	10/10^*b*^
Δ*proBAC*	10/10^*b*^	10/10^*b*^

a Mice were s.c. inoculated with 10^8^ CFU of the mutant strains. Mice were challenged with 100 LD_50_ (50% lethal dose) of the Fujisawa strain on day 14 after immunization and observed for 14 days. NA, not applicable.

b *P* < 0.01 compared to the respective control group by Fisher’s exact test.

### Virulence and vaccine efficacy of gene deletion mutants in pigs.

The virulence and vaccine efficacy of the proline biosynthesis mutants were further analyzed in pigs by subcutaneously inoculating them with the mutants. The main clinical signs of swine erysipelas include erythema, and pigs usually exhibit erythema at the site of inoculation with live attenuated vaccines (13). Pigs inoculated with 10^8^ CFU of Δ*proC* showed no skin reaction at the inoculation site, indicating high attenuation of the Δ*proC* mutant. In contrast, all pigs inoculated with Δ*proA*, Δ*proB*, Δ*proBA*, or Δ*proBAC* exhibited a skin reaction at the inoculation site; additionally, one pig each from the Δ*proA*- and Δ*proBA*-inoculated groups developed erythema at a few other body sites, and another pig from the *proBA*-inoculated group developed systemic erythema. In these pigs, the erythema disappeared by day 8 after inoculation. In the vaccine efficacy assay, the pigs were challenged with a lethal dose of the parent Fujisawa strain. The results showed that in contrast to the control pigs, all pigs inoculated with Δ*proA*, Δ*proB*, Δ*proC*, Δ*proBA*, or Δ*proBAC* were protected against the challenge infection; the pigs immunized with Δ*proC* developed erythema at a few body sites in addition to the challenge inoculation site, whereas the pigs immunized with other mutants showed no skin reaction. The agglutinating IgG antibody titers measured after vaccination are shown in Table S1. Overall, it was confirmed that all the mutants were safe for use, causing no systemic symptoms, and induced complete protection; however, no significant difference was achieved due to the small numbers of animals used.

Oral vaccination would be the ideal vaccination method in terms of cost of administration and animal welfare. The safety and vaccine efficacy via the oral route of the proline biosynthesis mutants in pigs were further evaluated using the Δ*proBAC* strain following single- or double-dose administration. After oral administration, the pigs in the single- and double-dose vaccination groups exhibited no signs of infection, showing normal body temperature, indicating that oral administration of the Δ*proBAC* strain at a dose of up to 10^10^ CFU was safe. After lethal infection with the parent Fujisawa strain, the control pigs developed typical signs of erysipelas and had to be euthanized by day 3 after challenge infection. The pigs in the vaccination groups produced agglutinating antibacterial antibodies and were completely protected without any clinical signs (*P < *0.01, control versus single-dose group; *P < *0.01, control versus double-dose group by Fischer’s exact test). In a postmortem analysis, *E. rhusiopathiae* bacteria were recovered from the tonsils of most pigs and from the hearts, lung, and mesenteric lymph of a few pigs. PCR analysis confirmed that all the colonies isolated from the organs were the Δ*proBAC* strain and that the challenge Fujisawa strain was not recovered. Experimental data on oral vaccination are shown in Tables S2 to S5.

## DISCUSSION

In this study, to rationally develop a live-attenuated vaccine against the genome-reduced pathogen *E. rhusiopathiae*, we employed “reverse vaccinology,” which uses genomic information, for the identification of genes that are specifically upregulated during the organism’s infectious processes. Sequence analysis of the whole genome of *E. rhusiopathiae* revealed that the organism possesses complete biosynthetic pathways for only seven amino acids (alanine, asparagine, glutamine, serine, cysteine, glycine, and proline) (14). We hypothesized that the genes involved in the biosynthesis of these amino acids must have escaped chromosomal deletion during evolution because of their importance for survival and pathogenicity and should therefore be upregulated *in vivo* during infection. We examined the mRNA expression levels of all genes involved in amino acid biosynthesis in this bacterium, which included 11 genes necessary for the biosynthesis of the seven amino acids and 3 additional genes involved in an incomplete biosynthetic pathway for arginine.

qRT-PCR analysis of the expression of the 14 genes revealed that compared to the expression levels during growth in medium without macrophages, significant upregulation was observed for 7 genes. Notably, the expression levels of the three genes *cysK*, *proC*, and *arcA* were very high. Considering that cysteine is the most limiting amino acid in mammalian cells and that pathogens have evolved to cope with the limited supply of cysteine within mammalian cells (16, 17), the high expression of *cysK* was not surprising. Rather, it was interesting that *E. rhusiopathiae* appeared to have evolved in a distinct way to synthesize this amino acid because many intracellular pathogens, including Listeria monocytogenes, Legionella pneumophila, Chlamydia trachomatis, and Rickettsia prowazekii, cannot synthesize cysteine and completely rely on their host cells to supply it (17). In contrast, the high expression levels observed for *arcA* and *arcC* were not expected because the arginine biosynthesis pathway was incomplete in *E. rhusiopathiae*. It has been shown that arginine metabolism and regulation are closely linked to the virulence of intracellular pathogens, including L. monocytogenes, L. pneumophila, Mycobacterium tuberculosis, and Mycobacterium bovis (20–24). For example, *arcA* has been shown to play a role in the growth of L. monocytogenes in acidic conditions (22). Taken together, these results suggest that the *arcA*, *arcC*, and *argF* genes, all of which are conserved in the incomplete arginine biosynthesis pathway of *E. rhusiopathiae*, may play important roles during macrophage infection. We found that of the 14 genes examined, 10 genes, excluding *sdaB*, *asnA*, *arcA*, and *arcC*, were conserved in Mycobacterium leprae (25), which has also undergone extensive reductive evolution. Thus, it was interesting that these two evolutionarily distant pathogens appeared to have evolved with conservation of the same amino acid biosynthesis genes for adaptation to nutritional environments within the host.

We hypothesized that the amino acid biosynthesis genes conserved in the genome-reduced bacteria may be the best targets for deletion and the construction of live-attenuated vaccines. However, gene deletion was unsuccessful for most of the amino acid biosynthesis genes of *E. rhusiopathiae*, suggesting that they are indispensable for its survival. Thus, it appears that our strategy cannot be applied to other genome-reduced bacteria because the genes selectively conserved after reductive evolution may be essential for the bacterium. However, there are many dispensable genes even in bacteria with highly reduced genomes, such as Mycoplasma genitalium (26). It has been shown that the intracellular pathogen *Francisella* possesses intact biosynthetic pathways for both glutamate and asparagine but still takes up these amino acids from infected cells (27). Furthermore, Salmonella has developed functionally redundant systems for the biosynthesis and transport of important amino acids for growth and virulence during evolution (28). Thus, many pathogens, including intracellular bacteria, rely on the transporter-mediated acquisition of amino acids from the host (17). Overall, it appears that in many bacteria, dispensable but important genes are functionally compensated by other systems. Notably, like mycoplasmas (29), *E. rhusiopathiae* possesses many transporters, with a high percentage (minimum 13.7%) of its genes devoted to transport functions (14), suggesting that *E. rhusiopathiae* can acquire proline from the environment. This may explain why it was possible to delete the *proB*, *proA*, and *proC* genes in the proline biosynthesis pathway.

We found that all the mutants generated in this study, including Δ*proA*, Δ*proB*, Δ*proC*, Δ*proBA*, and Δ*proBAC*, showed attenuated growth when incubated with J774A.1 cells. Microscopy observations revealed that in contrast to the proline gene deletion mutants, the parent Fujisawa strain replicated extensively both extracellularly and intracellularly during the infection of macrophages. The extra- and intracellular proliferation of the parent Fujisawa strain was expected; because it possesses a capsule, *E. rhusiopathiae* resists phagocytosis by macrophages, but even if phagocytized, it can replicate intracellularly (15). However, we found that in RPMI-FBS alone, the live bacterium numbers of all the mutants increased with the same kinetics observed in the parent Fujisawa strain, supporting our contention that the proline gene deletion mutants acquired proline from the environment. It remains unclear why the proline gene deletion mutants that attached to the surface of macrophages could not proliferate; however, it may be possible that under the applied experimental conditions, *de novo* synthesis of proline is preferred to proline uptake from the medium and that the cell-attached *E. rhusiopathiae* Fujisawa strain extracted nutrients directly from the macrophages in a contact-dependent manner (30), resulting in efficient growth conditions. This possibility needs to be investigated.

It was found that Δ*proC* was the most attenuated in mice *in vivo* and was the least immunogenic in pigs. In our previous study (13), we confirmed that a transposon mutant of the ERH_0056 gene, which was located immediately downstream of *proC* (ERH_0057), was as highly virulent as the parent strain, showing that inactivation of ERH_0056 gene does not affect *E. rhusiopathiae* virulence. This finding excludes the possibility that high attenuation of Δ*proC* was due to polar effects caused by the deletion of *proC*. Thus, the differences between virulence and immunogenicity among the mutants are unknown. However, it is possible that, similar to findings in Salmonella enterica (31), the severe attenuation of Δ*proC* may be due to the harmful accumulation of Δ^1^-pyrroline-5-carboxylate, a metabolic intermediate that is reduced to proline by ProC. Importantly, we observed that among the studied *pro* genes, the *proC* gene was the most upregulated during the infection of J774A.1 cells. The accumulation of proline and its oxidative metabolism are major mechanisms underlying organismal protective effects against a wide range of stresses, including hydrogen peroxide, nitric oxide, and osmotic stresses (32, 33). Despite its genome reduction, *E. rhusiopathiae* has evolved redundant defense mechanisms against reactive oxygen species (ROS) (14, 15, 34), suggesting that the avoidance or suppression of ROS-mediated killing by phagocytic cells may be the most important virulence strategy of *E. rhusiopathiae*. Similar to the essential role of *proC* in the virulence of M. tuberculosis (35), *E. rhusiopathiae proC* may play a critical role in protecting the organism against ROS stress.

In conclusion, we examined the mRNA expression of amino acid biosynthetic genes that remained conserved after the reductive evolution of *E. rhusiopathiae* and found that half of the 14 genes tested were upregulated during macrophage infection. Among the tested genes, we were able to delete only 3 genes involved in the proline biosynthesis pathway. It was confirmed that proline gene deletion mutants were attenuated and induced protective immunity in mice and pigs. Thus, our approach could significantly reduce the time necessary for the identification of important genes which are specifically upregulated during infection. It is possible that this approach will aid in the development of safe and effective live-attenuated vaccines against not only genome-reduced bacteria but also bacteria with moderate genome sizes, including Streptococcus, *Bartonella*, and *Clostridium* species, many of which are deficient in amino acid biosynthesis (36).

## MATERIALS AND METHODS

### Bacterial strains and media.

The *E. rhusiopathiae* strains used in this study included the wild-type Fujisawa strain, which was originally isolated from a septicemic pig, and mutant derivatives of this strain (Table 2). *E. rhusiopathiae* strains were grown at 37°C in brain heart infusion broth (Becton, Dickinson and Co., Baltimore, MD) supplemented with 0.1% Tween 80 and 0.3% Tris (pH 8.0) (BHI-T80). In pig experiments, to meet the requirements for biological products that are required to contain minimal animal-derived products, the *E. rhusiopathiae* strains were cultured in trypticase soy broth (Becton, Dickinson and Co.) supplemented with 0.1% Tween 80 (TSB-T80).

**TABLE 2:** *E. rhusiopathiae* strains used in this study^*a*^

Strain	Description
Fujisawa	Wild-type strain
Δ*proA*	In-frame deletion from nt 31–1,173 of ERH_0055 (31–1,173del)
Δ*proB*	In-frame deletion from nt 31–792 of ERH_0054 (31–792del)
Δ*proC*	In-frame deletion from nt 31–714 of ERH_0057 (31–714del)
Δ*proBA*	In-frame deletion from nt 31 of ERH_0054 to nt 1173 of ERH_0055
Δ*proBAC*	In-frame deletions from nt 31 of ERH_0054 to nt 1173 of ERH_0055 and nt 31–714 of ERH_0057

a nt, nucleotides; ERH_0055, glutamate-5-semialdehyde dehydrogenase; ERH_0054, glutamate 5-kinase; ERH_0057, pyrroline-5-carboxylate reductase.

### Real-time qRT-PCR.

The Fujisawa strain cultured in BHI-T80 at 37°C overnight was collected by centrifugation, washed, and resuspended in 10 mL of RPMI-FBS. Murine macrophage J774A.1 cells (10^6^) were incubated with the bacteria (10^8^ CFU; multiplicity of infection [MOI] = 100) at 37°C with rotation for 1 h in 10 mL of RPMI-FBS and then washed three times in ice-cold RPMI-FBS by vortex mixing and centrifugation at 900 rpm for 10 min to remove as many non-phagocytosed bacteria as possible. After washing, the samples were further incubated at 37°C with rotation for 6 h and then chilled on ice immediately after incubation. Macrophages were washed with ice-cold RPMI-FBS by vortex mixing and centrifugation and then pelleted. Bacterial cells suspended in RPMI-FBS containing no macrophage cells were used as a control. The bacterial cells in the control sample were pelleted by centrifugation at 12,000 rpm for 5 min at 4°C. Next, 200 μL of preheated Max Bacterial Enhancement Reagent (Invitrogen, Waltham, MA) was added to the macrophage and bacterial cell samples, and the samples were incubated at 95°C for 4 min. After incubation, 1 mL of TRIzol (Thermo Fisher Scientific, Waltham, MA) was added to the samples, and total RNA was isolated according to the manufacturer’s instructions. Contaminating genomic DNA was removed by treatment with RQ1 RNase-free DNase (Promega, Madison, WI), and RNA was reverse transcribed using an iScript Advanced cDNA Synthesis kit for RT-qPCR (Bio-Rad, Hercules, CA). Using the resulting cDNA as a template, the amino acid biosynthesis genes and *23S rRNA* were amplified using SsoAdvanced Universal SYBR Green Supermix (Bio-Rad) with the primers listed in Table S6. The relative expression levels of the target genes were normalized to the *23S rRNA* level using the comparative threshold cycle method.

### Construction of gene deletion mutants.

The construction of proline biosynthetic gene deletion mutants was performed as previously described (13, 37) with the following modifications. Briefly, the PCR primers shown in Table S6 were designed to include 15-bp extensions that could efficiently fuse DNA fragments and/or linearized vectors. The *proB* and *proA* genes are separated by 9 bp, suggesting that the *proBA* genes constitute an operon, while the *proC* gene is distant from the *proBA* genes on the chromosome (Fig. S2). Using genomic DNA of the *E. rhusiopathiae* Fujisawa strain as a template, the 5′- and 3′-flanking regions of the target gene were amplified by PCR with the primer sets proB-Up-F/proB-Up-R and proB-Do-F/proB-Do-R for the *proB* gene, proA-Up-F/proA-Up-R and proA-Do-F/proA-Do-R for the *proA* gene, and proC-Up-F/proC-Up-R and proC-Do-F/proC-Do-R for the *proC* gene. The fragments of the 5′- and 3′-flanking regions were cloned into the BglII and SalI sites of pMAD (38) using an In-Fusion HD cloning kit (Clontech Laboratories, Mountain View, CA) according to the manufacturer’s instructions. For the construction of the Δ*proBA* mutant, the 5′-flanking region of the *proB* gene and the 3′-flanking region of the *proA* gene were amplified by PCR using the primer sets proB-Up-F/proAB-R and proAB-F/proA-Do-R, respectively. These PCR products were cloned into pMAD as described above. The Δ*proBAC* mutant was generated from the Δ*proBA* strain by deleting the *proC* gene. In recombinant transformants, the deletion of the targeted gene(s) was confirmed by direct sequencing with an ABI PRISM 3130XL genetic analyzer (Applied Biosystems, Foster City, CA) using a BigDye Terminator v3.1 Cycle Sequencing kit (Applied Biosystems) according to the manufacturer’s instructions.

### Bacterial proliferation during the infection of murine macrophages.

The proliferation of *E. rhusiopathiae* strains treated with murine macrophage J774A.1 cells was assayed as previously described (34) with some modifications. Because *E. rhusiopathiae* is naturally resistant to gentamicin, which does not penetrate the cell membrane and is therefore used to kill extracellular bacteria, extracellular bacteria associated with macrophages were carefully removed by vortex mixing and centrifugation. Briefly, bacteria (10^7^ CFU) were incubated with macrophages (10^6^) (MOI = 10) in a total volume of 1.0 mL of RPMI-FBS under rotation (8 rpm) for 20 min at 37°C. The cells were washed three times with ice-cold RPMI-FBS by vortex mixing and centrifugation at 900 rpm for 10 min, and non-phagocytosed bacteria were removed. The cells containing internalized bacteria were resuspended in RPMI-FBS and then incubated at 37°C under rotation. After various intervals, a 0.1-mL sample was removed, serially diluted in Dulbecco’s phosphate-buffered saline (PBS) containing 0.5% Tween 20 and 5% FBS, and plated on BHI-T80 agar to determine the number of viable bacteria. Bacterial proliferation was expressed as a fold change normalized against the initial number of viable bacteria.

For microscopy observations, *E. rhusiopathiae* strains were incubated with J774A.1 cells (MOI = 1) in 35-mm glass-bottomed dishes (Matsunami Glass Ind., Ltd., Tokyo, Japan) for 1 h at 37°C in a 5% CO_2_ atmosphere. After incubation, the cells on the glass-bottom surface were carefully washed twice with 2 mL of PBS stored at room temperature (RT; 20 to 25°C) and then incubated at 37°C for 16 h in RPMI-FBS. After incubation, the cells were carefully washed twice with 3 mL PBS. The cells were fixed with 4% paraformaldehyde in PBS for 15 min at RT, permeabilized with PBS containing 0.4% Triton X-100 for 15 min at RT, and then treated with PBS containing 10% FBS for 30 min to block nonspecific binding of antibodies. For the detection of *E. rhusiopathiae* bacteria, the cells were incubated with primary rabbit antibodies against SpaA, a major cell surface protein (39). Bound antibodies were detected by incubation with secondary antibodies conjugated to Alexa 546 (Molecular Probes, Eugene, OR). Cell nuclei and F-actin were stained with DAPI (4′,6-diamidino-2-phenylindole) solution (Wako, Tokyo, Japan) and Alexa Fluor 488 phalloidin (Thermo Fisher Scientific, Waltham, MA), respectively. Images were captured on a BZ-X810 (Keyence, Osaka, Japan) at ×60 or ×100 magnification.

### Animal experiments.

Animal experiments were performed according to the regulations and guidelines of the Animal Ethics Committee of the National Institute of Animal Health (NIAH) in Tsukuba (Ibaraki, Japan). For mouse experiments, 6- to 8-week-old female ddY mice purchased from Japan SLC, Inc. (Hamamatsu, Shizuoka, Japan) were used throughout the experiments. Pigs were purchased from the Zen-Noh Central Research Institute for Feed and Livestock and used at the age of 4 weeks.

### Growth of proline biosynthesis mutants in mice.

Mice were inoculated subcutaneously (s.c.) with 0.1 mL of a bacterial suspension (10^9^ CFU/mL). On days 1, 2, 4, and 7 postinoculation, the mice were euthanized, and their spleens and livers were removed. The tissues were homogenized in BHI-T80, diluted, and plated onto selective BHI-T80 plates containing 0.001% crystal violet, 0.03% sodium azide, 400 μg/mL kanamycin, and 25 μg/mL gentamicin. After incubation for 48 h at 37°C, bacterial colonies were counted.

### Protection assay in mice.

To examine the protection ability of proline biosynthesis mutants, groups of 10 mice were s.c. immunized with 10^8^ CFU of the strains. Two weeks after immunization, mice were challenged with 10^3^ CFU (100 LD_50_ of the Fujisawa strain) of Fujisawa and observed for 14 days to record clinical symptoms and death.

### Attenuation and protection assay in pigs.

To examine whether the proline biosynthesis mutants were attenuated in a natural host, groups of pigs were s.c. inoculated with approximately 10^8^ CFU of the strains and observed for clinical symptoms. To further investigate the protective abilities of the strains, all inoculated pigs were challenged with 3 × 10^8^ CFU of Fujisawa 2 weeks after inoculation and observed for 12 days to record clinical symptoms and death.

Vaccine efficacy in pigs was further examined following the oral administration of the Δ*proBAC* strain. The vaccination tests were independently conducted with single- or double-dose inoculation at different times. In both tests, three pigs used as controls were fed either artificial milk for humans (Meiji Hohoemi RakuRakuMilk, Tokyo, Japan) or milk replacer for pigs (SPF-LAC; Weyerhaeuer, Eaton, OH), whereas the pigs in the vaccination groups were allowed to freely consume either artificial milk or milk replacer mixed with a bacterial suspension in a plastic container once (single-dose) or twice on 2 consecutive days (double-dose). Assuming that each pig consumed 10 mL of milk, the theoretical vaccination dose was 1.0 × 10^10^ CFU of the strain per pig per day. The pigs were s.c. challenged with 1.0 × 10^8^ CFU of the Fujisawa strain 21 days after vaccination and monitored for clinical signs and death for 14 days. Blood was periodically collected from the pigs during the experimental period to assess the systemic humoral immune response, which was examined by a growth agglutination test (13). This test is the most appropriate method for the detection of protective IgG antibodies against *E. rhusiopathiae* (13).

At necropsy, samples of blood, tissues, and organs (tonsils, hearts, lungs, livers, kidneys, spleens, mesenteric lymph nodes, knee joint cavities, and elbow joint cavities) were examined for the presence of *E. rhusiopathiae* bacteria by plating on selective BHI-T80 plates. The colonies on the selective plates were later checked (3 to 43 colonies per sample) in a PCR assay with the primers Proline-F and Proline-R to distinguish the Δ*proBAC* strain and the challenge Fujisawa strain.
