# Supplementary material for: Rational Design of Live-Attenuated Vaccines against Genome-Reduced Pathogens
Source: Microbiol Spectr. 2022 Dec 1;10(6):e03776-22. doi: 10.1128/spectrum.03776-22 (PMC9769512; doi:10.1128/spectrum.03776-22)
Supplement: Supplemental file 1 — Supplemental material. Download spectrum.03776-22-s0001.pdf, PDF file, 0.4 MB [file spectrum.03776-22-s0001.pdf]

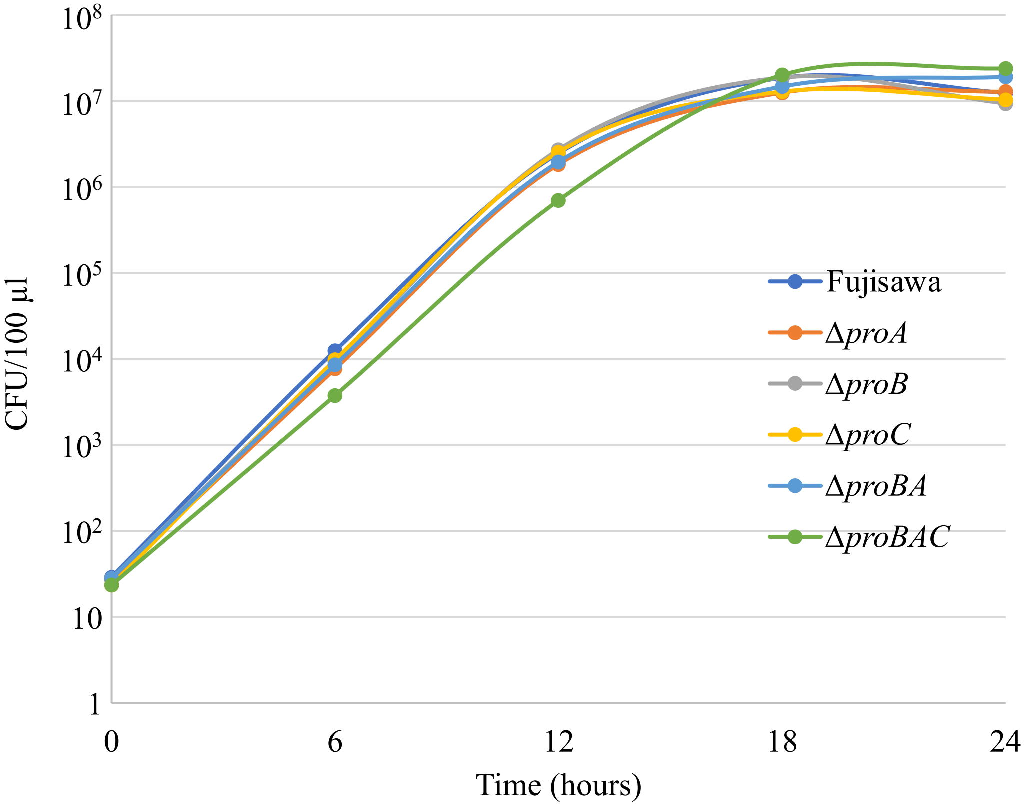


Supplemental Figure 1. Growth of *Erysipelothrix rhusiopathiae* Δ*proA*, Δ*proB*, Δ*proC,* Δ*proBA,* and Δ*proBAC* in RPMI-FBS. The bacterial counts at the zero time point were 29, 28, 28, 24, 28, and 24 colony forming units per 100 µl of RPMI-FBS for Fujisawa, Δ*proA*, Δ*proB*, Δ*proC*, Δ*proBA*, and Δ*proBAC*, respectively. The results are representative of three separate experiments .

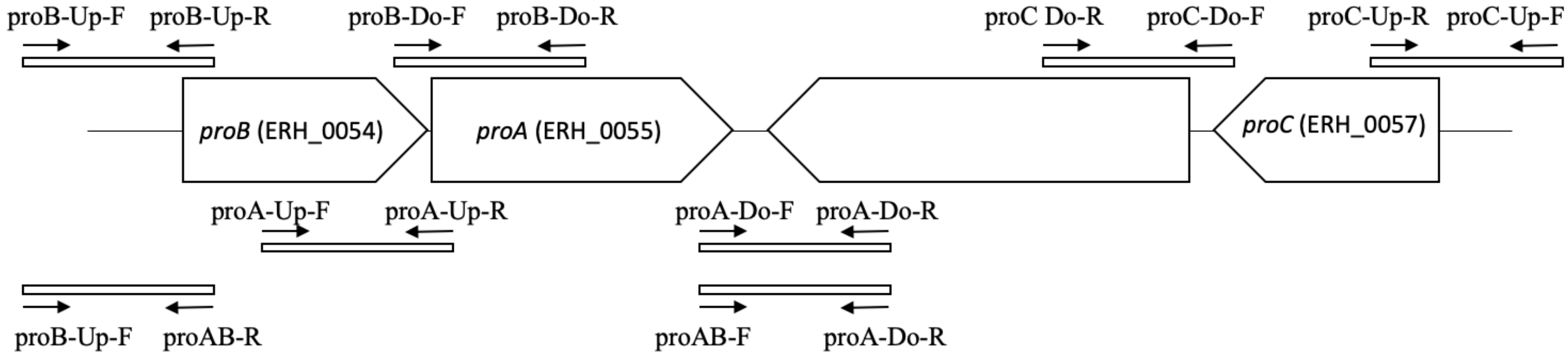


Supplemental Figure 2. Genetic organization of the *proA*, *proB*, and *proC* regions. The arrows indicate the orientation and corresponding locations of the primers used (Supplemental Table 6). The genes and PCR fragments shown are not to scale .

Supplemental Table 1. Agglutinating IgG antibody titers after subcutaneous inoculation of proline biosynthetic mutants in pigs[a]

| Pig group | Pig No. | Agglutinating IgG antibody titer[b] | | | | |
|---|---|---|---|---|---|---|
| | | Before vaccination | 7 days after vaccination | 14 days after vaccination | 7 days after challenge | 12 days after challenge |
| Non inoculated | 1 | <4 | <4 | <4 | -[c] | - |
| | 2 | <4 | <4 | <4 | - | - |
| Δ*proA* inoculated | 1 | <4 | <4 | 4 | 32 | 32 |
| | 2 | <4 | <4 | 4 | 128 | 128 |
| | 3 | <4 | <4 | 4 | 32 | 32 |
| Δ*proB* inoculated | 1 | <4 | <4 | <4 | 4 | <4 |
| | 2 | <4 | <4 | 4 | 32 | 32 |
| | 3 | <4 | <4 | 4 | 8 | 8 |
| Δ*proC* inoculated | 1 | <4 | <4 | 4 | 8 | 32 |
| | 2 | <4 | <4 | 4 | 16 | 16 |
| | 3 | <4 | <4 | 4 | 4 | 32 |
| Δ*proBA* inoculated | 1 | <4 | <4 | 64 | 128 | 64 |
| | 2 | <4 | <4 | 64 | 256 | 128 |
| | 3 | <4 | <4 | 128 | 256 | 128 |
| Δ*proBAC* inoculated | 1 | <4 | <4 | <4 | <4 | <4 |
| | 2 | <4 | <4 | 32 | 32 | 32 |
| | 3 | <4 | <4 | <4 | 8 | 16 |

[a] Pigs were inoculated subcutaneously with $10^8$ CFU of the strains and challenged with $3x10^8$ CFU of the Fujisawa strain on Day 14 after vaccination.

[b] The agglutination titer was determined as described previously (13). Briefly, serum samples were pretreated with 2-mercaptoethanol (2-ME) at 37 °C for 1 hour, subjected to two-fold serial dilution, and mixed with an equal volume of an overnight culture of *E. rhusiopathiae* Marienfelde diluted 1:10 with culture medium. The agglutination titer was determined after overnight incubation at 37 °C. The results are expressed as the reciprocal of the highest serum dilution showing agglutination.

[c] -, not applicable.

Supplemental Table 2. Agglutinating IgG antibody titers after oral vaccination of the Δ*proBAC* strain in pigs[a]

| Pig group | Pig No. | Agglutinating IgG antibody titer[b] | | | | | |
|---|---|---|---|---|---|---|---|
| | | Before vaccination | 7 days after vaccination | 14 days after vaccination | 21 days after vaccination | 7 days after challenge | 14 days after challenge |
| Non-vaccinated | 1 | <4 | <4 | <4 | <4 | -[c] | - |
| | 2 | <4 | <4 | <4 | <4 | - | - |
| | 3 | <4 | <4 | <4 | <4 | - | - |
| Orally vaccinated (single-dose) | 1 | <4 | 8 | 256 | 128 | 128 | 128 |
| | 2 | <4 | 4 | 64 | 64 | 64 | 64 |
| | 3 | <4 | 16 | 128 | 128 | 128 | 128 |
| | 4 | <4 | 4 | 256 | 128 | 128 | 128 |
| | 5 | <4 | 8 | 128 | 128 | 128 | 128 |
| | 6 | <4 | <4 | 8 | 8 | 16 | 16 |
| | 7 | <4 | 8 | 128 | 128 | 128 | 128 |
| | 8 | <4 | <4 | 256 | 128 | 128 | 128 |
| | 9 | <4 | 4 | 128 | 128 | 64 | 64 |
| | 10 | <4 | <4 | 8 | 16 | 32 | 32 |
| | 11 | <4 | <4 | 128 | 64 | 64 | 64 |

[a] Pigs were orally inoculated with $10^{10}$ CFU of the strains in artificial milk once and challenged with $10^{8}$ CFU of the Fujisawa strain on Day 21 after vaccination.

[b] The agglutination titer was determined as described previously (13). Briefly, serum samples were pretreated with 2-ME at 37 °C for 1 hour, subjected to two-fold serial dilution, and mixed with an equal volume of an overnight culture of *E. rhusiopathiae* Marienfelde diluted 1:10 with culture medium. The agglutination titer was determined after overnight incubation at 37 °C. The results are expressed as the reciprocal of the highest serum dilution showing agglutination.

[c] -, not applicable.

Supplemental Table 3. Recovery of *E. rhusiopathiae* from pigs following oral vaccination (single-dose)

| Pig No. | No. of bacteria isolated from ($\log_{10}$ CFU/g) (No. of colonies PCR-positive for Δ*proBAC* strain/No. of colonies tested) | |
|---|---|---|
| | Tonsil | Heart |
| 1 | 6.27 (10/10) | - [a] |
| 2 | 4.49 (14/14) | 3.67 (21/21) |
| 3 | 6.50 (12/12) | 2.29 (1/1) |
| 4 | 5.70 (12/12) | - |
| 5 | 5.33 (7/7) | - |
| 6 | - | - |
| 7 | 5.82 (28/28) | - |
| 8 | 5.38 (14/14) | - |
| 9 | 5.93 (41/41) | - |
| 10 | - | - |
| 11 | 3.40 (12/12) | - |

[a] Not recovered. Bacteria were also not recovered from blood, lung, liver, kidney, spleen, mesenteric lymph node, knee joint cavity, or elbow joint cavity samples.

Supplemental Table 4. Agglutinating IgG antibody titers after oral vaccination of the Δ*proBAC* strain in pigs[a]

| Pig group | Pig No. | Agglutinating IgG antibody titer[b] | | | | | |
|---|---|---|---|---|---|---|---|
| | | Before vaccination | 7 days after vaccination | 14 days after vaccination | 21 days after vaccination | 7 days after challenge | 14 days after challenge |
| Non-vaccinated | 1 | <4 | <4 | <4 | <4 | - [c] | - |
| | 2 | <4 | <4 | <4 | <4 | - | - |
| | 3 | <4 | <4 | <4 | <4 | - | - |
| Orally vaccinated (double-dose) | 1 | <4 | 16 | 16 | 16 | 32 | 32 |
| | 2 | <4 | 32 | 32 | 16 | 64 | 32 |
| | 3 | <4 | 8 | 32 | 16 | 16 | 16 |
| | 4 | <4 | 32 | 64 | 32 | 32 | 32 |
| | 5 | <4 | 8 | 16 | 32 | 32 | 32 |
| | 6 | <4 | 16 | 8 | 32 | 32 | 32 |
| | 7 | <4 | 16 | 32 | 32 | 32 | 32 |
| | 8 | <4 | 8 | 32 | 16 | 32 | 32 |
| | 9 | <4 | 4 | 32 | 8 | 8 | 8 |
| | 10 | <4 | 32 | 32 | 8 | 8 | 16 |
| | 11 | <4 | 8 | 32 | 32 | 32 | 32 |
| | 12 | <4 | 32 | 16 | 8 | 16 | 16 |

[a] Pigs were inoculated orally with $10^{10}$ CFU of the strains in milk replacer for 2 consecutive days and challenged with $10^{8}$ CFU of the Fujisawa strain on Day 21 after the final vaccination.

[b] The agglutination titer was determined as described previously (13). Briefly, serum samples were pretreated with 2-ME at 37 °C for 1 hour, subjected to two-fold serial dilution, and mixed with an equal volume of an overnight culture of *E. rhusiopathiae* Marienfelde diluted 1:10 with culture medium. The agglutination titer was determined after overnight incubation at 37 °C. The results are expressed as the reciprocal of the highest serum dilution showing agglutination.

[c] -, not applicable.

Supplemental Table 5. Recovery of *E. rhusiopathiae* from pigs following oral vaccination (double-dose)

| Pig No. | No. of bacteria isolated from ($log_{10}$ CFU/g) (No. of colonies PCR-positive for Δ*proBAC* strain/No. of colonies tested) | | | |
|---|---|---|---|---|
| | Tonsil | Heart | Lung | Mesenteric lymph node |
| 1 | 6.71 (33/33) | - [a] | - | - |
| 2 | 6.67 (32/32) | - | - | - |
| 3 | 6.08 (8/8) | - | - | - |
| 4 | 6.65 (33/33) | - | - | - |
| 5 | 6.30 (14/14) | 2.65 (3/3) | - | - |
| 6 | 7.44 (7/7) | 4.27 (7/7) | 2.96 (3/3) | - |
| 7 | 6.55 (15/15) | - | - | - |
| 8 | 6.96 (43/43) | 3.39 (9/9) | - | - |
| 9 | 6.80 (33/33) | - | - | 3.11 (7/7) |
| 10 | 5.02 (14/14) | - | - | - |
| 11 | 5.31 (12/12) | - | - | - |
| 12 | 6.86 (42/42) | 3.01 (5/5) | - | - |

[a] Not recovered. Bacteria were also not recovered from blood, liver, kidney, spleen, knee joint cavity, or elbow joint cavity samples.

Supplemental Table 6. Primers used in this study.

| Primer | Sequences (5' to 3') | Description |
|---|---|---|
| 23S-rRNA-qRT-F | CCCGTGAGGAAGTAGCGAAG | Used for qRT–PCR assay to amplify *23S-rRNA* gene |
| 23S-rRNA-qRT-R | GGGGTCATTTTGCCGAGTT | Used for qRT–PCR assay to amplify *23S-rRNA* gene |
| ald-qRT-F | CGGATGTTGTGATTGGGAGT | Used for qRT–PCR assay to amplify *ald* gene |
| ald-qRT-R | CTGCACCTGGCATATTGGTT | Used for qRT–PCR assay to amplify *ald* gene |
| arcA-qRT-F | TGAAGAAGGCGGAATCAAAA | Used for qRT–PCR assay to amplify *arcA* gene |
| arcA-qRT-R | GCAAATGGGTCACGTGTAAAG | Used for qRT–PCR assay to amplify *arcA* gene |
| arcC-qRT-F | GGACGTGGGTATCGTCGTGT | Used for qRT–PCR assay to amplify *arcC* gene |
| arcC-qRT-R | CGGCATTTAGGTCAAGAGCA | Used for qRT–PCR assay to amplify *arcC* gene |
| argF-qRT-F | TGGACTTGGGTATGGGTGTT | Used for qRT–PCR assay to amplify *argF* gene |
| argF-qRT-R | TCTGCGATCATTTGTGTTGG | Used for qRT–PCR assay to amplify *argF* gene |
| asnA-qRT-F | ATGGTGTGGAGCGTCCTGTT | Used for qRT–PCR assay to amplify *asnA* gene |
| asnA-qRT-R | TCGAGGGTACGTTGTTCACG | Used for qRT–PCR assay to amplify *asnA* gene |
| cysE-qRT-F | ATTTCGCAGCTTGGACGATT | Used for qRT–PCR assay to amplify *cysE* gene |
| cysE-qRT-R | GTGTCGTTTCCCTGGCTCTC | Used for qRT–PCR assay to amplify *cysE* gene |
| cysK-qRT-F | TAACTGAAGCACGCAAGCAA | Used for qRT–PCR assay to amplify *cysK* gene |
| cysK-qRT-R | TCATGCCAGAAGCACCATC | Used for qRT–PCR assay to amplify *cysK* gene |
| glnA-qRT-F | AACCTGATCGAAACGGGAAA | Used for qRT–PCR assay to amplify *glnA* gene |
| glnA-qRT-R | AACATGGCCCCGAATAAATG | Used for qRT–PCR assay to amplify *glnA* gene |
| glyA-qRT-F | GCCATTGAATTGGAAGAGCA | Used for qRT–PCR assay to amplify *glyA* gene |
| glyA-qRT-R | CGACATACTCACAGCCACCA | Used for qRT–PCR assay to amplify *glyA* gene |

| | | |
|---|---|---|
| proA-qRT-F | GCCCTTAAACGTATTGGTGACG | Used for qRT–PCR assay to amplify *proA* gene |
| proA-qRT-R | TGCTTCTATGCGTGCTTGATTT | Used for qRT–PCR assay to amplify *proA* gene |
| proB-qRT-F | CCGCGTTTTGGCTGATTT | Used for qRT–PCR assay to amplify *proB* gene |
| proB-qRT-R | TAGCCATTTTATCGGGGATTTG | Used for qRT–PCR assay to amplify *proB* gene |
| proC-qRT-F | TATGAGACCGCCCTTAGAATTACC | Used for qRT–PCR assay to amplify *proC* gene |
| proC-qRT-R | ACCAGGGGAACAAACACGA | Used for qRT–PCR assay to amplify *proC* gene |
| sdaA-qRT-F | TCAAAATGCGGTAGGAACCA | Used for qRT–PCR assay to amplify *sdaA* gene |
| sdaA-qRT-R | CACCAAACCAAACCCTCCA | Used for qRT–PCR assay to amplify *sdaA* gene |
| sdaB-qRT-F | TAGCGGGAGGTCTTTTAGGG | Used for qRT–PCR assay to amplify *sdaB* gene |
| sdaB-qRT-R | ATTCCAACAACCGTGCATTT | Used for qRT–PCR assay to amplify *sdaB* gene |
| proA-Up-F | TAACTAGACAGATCTGTTGTGCTTGTGTCTTCTG | Used for construction of the Δ*proA* strain |
| proA-Up-R | ACCCTTTCCTTTTACTTCTTTTGCCCTTATTCCAATGTCTTTCAT | Used for construction of the Δ*proA* strain |
| proA-Do-F | ATAAGGGCAAAAGAAGTAAAAGGAAAGGGTCAGACACGTGTCTGA | Used for construction of the Δ*proA* strain |
| proA-Do-R | CCATATGACGTCGACGGACTTACAACCTACTTCACCTCGC | Used for construction of the Δ*proA* and Δ*proBA* strains |
| proB-Up-F | TAACTAGACAGATCTGGATCAATAGATTGGAGTTTTCC | Used for construction of the Δ*proB* and Δ*proBA* |
| proB-Up-R | TTCCTCCTATAAATATTTTTATCTCTCAAAACTTCTCTATTCAT | Used for construction of the Δ*proB* strain |

| | | |
|---|---|---|
| proB-Do-F | TTGAGAGATAAAAAATATTTATAGGAGGAACGATGAAAG | Used for construction of the Δ*proB* strain |
| proB-Do-R | CCATATGACGTCGACCATCGATACATCCGCATCATAGTC | Used for construction of the Δ*proB* strain |
| proC-Up-F | CCATATGACGTCGACCTTTACCGATAACAATATCACTCCTTTCTCTG | Used for construction of the Δ*proC* and Δ*proBAC* strains |
| proC-Up-R | TTTAAGAATTGTAGCATTACCAACGCCGATAAATCCAATTTTCAT | Used for construction of the Δ*proC* and Δ*proBAC* strains |
| proC-Do-F | ATCGGCGTTGGTAATGCTACAATTCTTAAAGATAAACAAGGATAA | Used for construction of the Δ*proC* and Δ*proBAC* strains |
| proC-Do-R | TAACTAGACAGATCTAGCAGTAATTACTTCGCGACGGTG | Used for construction of the Δ*proC* and Δ*proBAC* strains |
| proAB-F | TTGAGAGATAAAAAAGTAAAGGAAAGGGTCAGACACGTGTCTGA | Used for construction of the Δ*proBA* strain |
| proAB-R | ACCCTTTCCTTTTACTTTTTTATCTCTCAAAACTTCTCTATTCAT | Used for construction of the Δ*proBA* strain |
| Proline-F | GCAGCGAATTGTAAACTGTTGTTTCCAG | Forward primer used for differentiation of the Δ*proBAC* strain and the challenge Fujisawa strain |
| Proline-R | CTTTACCGATAACAATATCACTCCTTTCTCTG | Reverse primer used for differentiation of the Δ*proBAC* strain and the challenge Fujisawa strain |

Underlined sequences are 15-bp extensions that are complementary to the ends of PCR-generated fragments or linearized vectors.
